# Characterization of proteome alterations in *Phanerochaete chrysosporium *in response to lead exposure

**DOI:** 10.1186/1477-5956-9-12

**Published:** 2011-03-09

**Authors:** Volkan Yıldırım, Servet Özcan, Dörte Becher, Knut Büttner, Michael Hecker, Gülay Özcengiz

**Affiliations:** 1 Department of Biological Sciences, Middle East Technical University, Ankara, Turkey; 2 Department of Biology, Erciyes University, Kayseri, Turkey; 3 Institut für Mikrobiologie, Ernst-Moritz Arndt-Universität Greifswald, Greifswald, Germany

## Abstract

**Background:**

Total soluble proteome alterations of white rot fungus *Phanerochaete chrysosporium *in response to different doses (25, 50 and 100 μM) of Pb (II) were characterized by 2DE in combination with MALDI-TOF-MS.

**Results:**

Dose-dependent molecular response to Pb (II) involved a total of 14 up-regulated and 21 down-regulated proteins. The induction of an isoform of glyceraldehyde 3-phosphate dehydrogenase, alcohol dehydrogenase class V, mRNA splicing factor, ATP-dependent RNA helicase, thioredoxin reductase and actin required a Pb (II) dose of at least 50 μM. Analysis of the proteome dynamics of mid-exponential phase cells of *P. chrysosporium *subjected to 50 μM lead at exposure time intervals of 1, 2, 4 and 8 h, identified a total of 23 proteins in increased and 67 proteins in decreased amount. Overall, the newly induced/strongly up-regulated proteins involved in (i) amelioration of lipid peroxidation products, (ii) defense against oxidative damage and redox metabolism, (iii) transcription, recombination and DNA repair (iv) a yet unknown function represented by a putative protein.

**Conclusion:**

The present study implicated the particular role of the elements of DNA repair, post-tanscriptional regulation and heterotrimeric G protein signaling in response to Pb (II) stress as shown for the first time for a basidiomycete.

## Background

Heavy metal pollution is a major environmental concern due to its toxic effects through the food chain and its high persistence in the environment [1]. Lead (Pb) is one of the most abundant toxic metal; mining and smelting activities, lead containing paints, paper and pulp, gasoline and explosives as well as the disposal of municipal sewage sludge enriched with Pb being the main sources of pollution [2]. Possible mechanisms involved in metal-induced oxidative stress were extensively reviewed by Ercal *et al. *[3]. As a redox-inactive metal, Pb is known to deplete cells' major antioxidants, thiol-containing antioxidants and enzymes in particular.

The unique oxidative enzyme system of white-rot basidiomycetes is directly involved in complete lignin mineralization and degradation of various xenobiotic compounds as well as dyes [4,5]. *Phanerochaete chrysosporium *is one of the best studied white-rot fungi shown to be very promising for treatment of phenolic effluents from pulp and paper, coal conversion, textile and olive oil industries. This organism is also very effective in biosorbing heavy metal ions from dilute solutions [6-8], with an equilibrium adsorptive capacity order of lead (II) > chromium (III) > copper (II) = cadmium (II) > nickel (II) [9]. Recently, *P*. *chrysosporium *was successfully employed for bioremediation of lead-contaminated soil [10,11]. It is well-known that tolerance to different metals varies greatly among microorganisms. When the above-mentioned heavy metals were compared for their effects on growth of *P*. *chrysosporium*, lead was the best tolerated metal in that the concentrations up to 100 μM did not interfere with growth in liquid cultures (unpublished). Comparatively, only 5 μM of this metal inhibits the growth of *S*. *cerevisiae *by approximately 30% [12].

The concentration level of lead found in wastewaters varies greatly depending on the type of wastewater which is ranging between 0.04 to 0.05 ppm in urban wastewater [13,14], 0.06 to 2.60 ppm in industrial wastewater [15], 1.27 ppm in metal finishing waste water in particular [16], and 20 to 100 ppm in waste biogas residual slurry [17]. Temporal variability of these concentrations as caused, for example, by rain events or regular daily fluctuations as seen in wastewater treatment plant effluents is to be noted [18].

While the effects of toxic metals on cell physiology can be studied at the level of individual proteins, proteomics has allowed the responses to be studied on a much wider scale [19,20]. Regarding eukaryotic microorganisms, heavy metal stress proteomics have been published for *S. cerevisiae *[21,22], *Schizosaccharomyces pombe *[23] and *Chlamydomonas reinhardtii *[24]. Our group reported the first reference proteome map of *P*. *chrysosporium *along with an analysis of cadmium and copper response in this organism [25]. A total of 80 Cd-up-regulated and 74 Cu-up-regulated protein spots were detected and identified, 34 being common to the stress caused by both metals. Thus the aim of the present study is to determine the dynamic response of *P. chrysosporium *proteome to subtoxic levels of lead.

## Results and Discussion

To investigate the proteome response of *P. chrysosporium *to Pb (II) exposure we used 2D electrophoresis followed by MALDI-TOF analysis. The cells were exposed either three levels of Pb (25, 50 and 100 μM) for 40 h or 50 μM of Pb after 40 h incubation for 1, 2, 4 and 8 h. This kind of setup helped us to observe proteome response of *P. chrysosporium *to acute and chronic exposure of Pb (II). The highest lead concentration employed in this study (100 μM; ca 20 ppm) is about 8 fold higher than its upper level reported for industrial wastewaters [15].

When subjected to different levels of Pb (II), a total of 14 up-regulated and 21 down-regulated protein spots were identified. (Table 1 and 2). Among the up-regulated proteins, the proteins common to all three dosages included isocitrate dehydrogenase alpha subunit, UDP-glucose pyrophosphorylase, septin family protein (P-loop GTPase), F0F1-type ATP synthase, alpha subunit, GTPase Ran/TC4/GSP1, short-chain acyl-CoA dehydrogenase, polyadenylate-binding protein (RRM superfamily) and G protein beta subunit-like protein. On the other hand, the induction of an isoform of glyceraldehyde 3-phosphate dehydrogenase, alcohol dehydrogenase class V, mRNA splicing factor, ATP-dependent RNA helicase, thioredoxin reductase and actin required a Pb (II) dose of at least 50 μM.

**Table 1:** Dose-dependent upregulated protein spots in response to Pb (II)

			Treatment/Control Ratio				
					
KOG Class	Protein ID	Putative Function	25 μM	50 μM	100 μM	Subcellular Locations*	Multiple Spots
**Amino acid transport and metabolism**	139320a	Isocitrate dehydrogenase, alpha subunit	5,14	5,02	4,06	Mit	2

**Carbohydrate transport and metabolism**	134115	UDP-glucose pyrophosphorylase	3,50	3,05	5,22	Cyto_nuc	-
	135471	Septin family protein (P-loop GTPase)	**New**	**New**	**New**	M	-
	132198d	Glyceraldehyde 3-phosphate dehydrogenase	-	**New**	**New**	C	4

**Cytoskeleton**	139298a	Actin and related proteins	1,38	11,01	1,43	Cysk	4

**Energy production and conversion**	137299d	F0F1-type ATP synthase, alpha subunit	**New**	**New**	**New**	Mit	4

**General function prediction only**	134635	mRNA splicing factor	2,29	4,62	2,26	C	-

Intracellular trafficking, secretion, and vesicular transport	123314	GTPase Ran/TC4/GSP1 (nuclear protein transport pathway), small G protein superfamily	**New**	**New**	**New**	C	-

**Lipid transport and metabolism**	1819	Short-chain acyl-CoA dehydrogenase	3,60	3,70	4,30	C	-

**Posttranslational modification, protein turnover, chaperones**	8527	Thioredoxin reductase	2,54	3,49	4,46	Mit	-

**RNA processing and modification**	123005a	Polyadenylate-binding protein (RRM superfamily)	**New**	**New**	**New**	Cysk	2
	126823	ATP-dependent RNA helicase	0,97	7,89	1,86	Nuc	-

**Secondary metabolites biosynthesis, transport and catabolism**	4796c	Alcohol dehydrogenase, class V	-	**New**	**New**	C	4

**Signal transduction mechanisms**	10373	G protein beta subunit-like protein	3,10	3,30	4,60	C	-

*C; cytoplasmic, Ext; extracellular, Mit; mitochondrial, Nuc; Nuclear, Cysk; cytoskeleton, Cyto_nucl; cytoplasmic_nuclear

**Table 2:** Dose-dependent downregulated protein spots in response to Pb (II)

			Treatment/Control Ratio				
					
KOG Class	Protein ID	Putative Function	25 uM	50 uM	100 uM	Subcellular location^*^	Multiple spots
**Amino acid transport and metabolism **	138721a	Glutamate/leucine/phenylalanine/valine dehydrogenases	0,50	0,22	1,32	C	2
	139320b	Isocitrate dehydrogenase, alpha subunit	0,12	0,02	0,01	Mit	3
	139663&138887a	Methionine synthase II (cobalamin-independent)-//-aconitate hydratase	0,24	0,09	0,08	C	4
	139663&138887b	Methionine synthase II (cobalamin-independent)-//-aconitate hydratase	0,30	0,06	0,15	C	4
	139663&138887c	Methionine synthase II (cobalamin-independent)-//-aconitate hydratase	0,14	0,00	0,05	C	4
	139663&138887d	Methionine synthase II (cobalamin-independent)-//-aconitate hydratase	0,26	0,18	0,06	C	4
	139663d	Methionine synthase II (cobalamin-independent)	0,15	0,17	0,66	C	6
	139663e	Methionine synthase II (cobalamin-independent)	0,20	0,20	0,60	C	6
	139663f	Methionine synthase II (cobalamin-independent)	0,07	0,02	0,01	C	6

**Cytoskeleton**	139298c	Actin and related proteins	0,59	0,16	1,15	Cysk	4

**Energy production and conversion**	1056	Zinc-binding oxidoreductase	0,14	0,09	0,25	C	-
	123932	Fumarate reductase, flavoprotein subunit	0,14	0,07	0,34	C	-
**Lipid transport and metabolism**	10015	Acetyl-CoA acetyltransferase	0,66	0,24	0,88	C	2

**NA**	8290	hypothetical protein	0,40	0,22	0,28	C	-
	140431b	hypothetical protein	0,04	0,03	0,01	C	4
	140431c	hypothetical protein	0,19	0,03	0,07	C	4
	140431d	hypothetical protein	0,52	0,22	0,11	C	4

**Posttranslational modification**,	122440	Molecular chaperones HSP70/HSC70, HSP70 superfamily	0,53	0,09	0,84	C	6
**protein turnover, chaperones**	131983b	Molecular chaperones GRP78/BiP/KAR2, HSP70 superfamily	0,71	0,11	0,19	Ext	2
**Secondary metabolites biosynthesis, transport and catabolism**	8565	Hydroxysteroid 17-beta dehydrogenase 11	0,45	0,29	1,24	Mit	-

**Signal transduction mechanisms**	10895	Glycosylphosphatidylinositol-specific phospholipase C	0,11	0,30	1,19	Cyto_ nucl	-

*C; cytoplasmic, Ext; extracellular, Mit; mitochondrial, Nuc; Nuclear, Cysk; cytoskeleton, Cyto_nucl; cytoplasmic_nuclear

**Figure 1:**
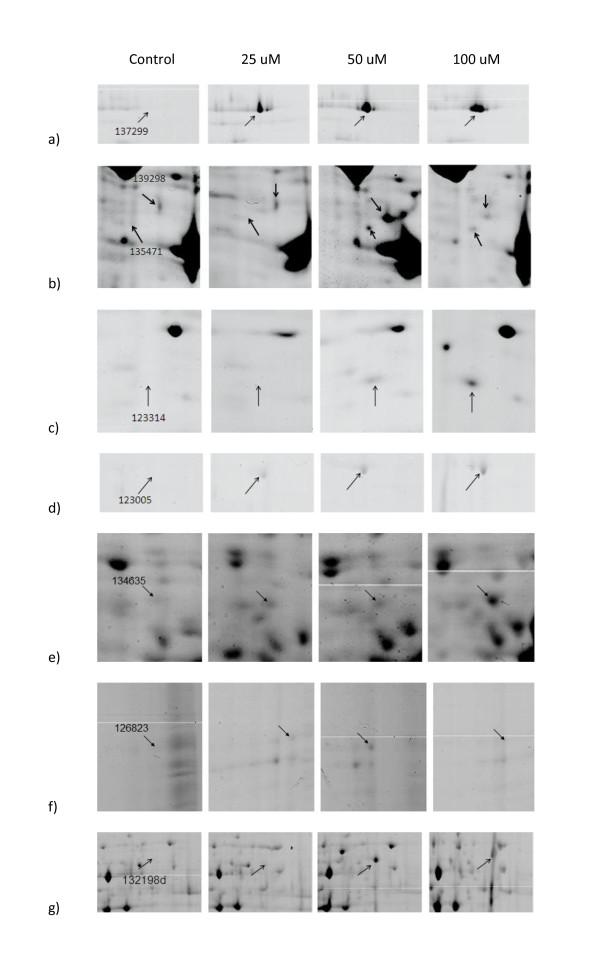
**Dose-dependent upregulated protein spots**.

Among the up-regulated proteins, 6 were newly induced. One such protein was mitochondrial F0F1-type ATP synthase, alpha subunit (Figure 1a) which pointed to galvanized energy metabolism under stress. The other one was **actin **(Figure 1b) constituting a cytoskeleton that plays a pivotal role in many eukaryotic signaling pathways. Actin-induced hyperactivation of the Ras signaling pathway was demonstrated to lead to apoptosis in *S. cerevisiae *[26]. A septin family protein (P-loop GTPase) was also newly induced in response to Pb (II) exposure (Figure 1b). Septin family of cytoskeletal proteins with GTPase activity are involved in many processes including membrane dynamics, vesicle trafficking, apoptosis and infection [27]. Ras GTPase [GTPase Ran/TC4/GSP1 (nuclear protein transport pathway; small G protein superfamily)] was found as a Cd responsive protein in our previous study [25], providing clue for the existence of Ras signaling pathway in *P. chrysosporium*, a pathway accelerating programmed cell death in *C. albicans *under harsh environmental stress [28]. In the present work, Ras GTPase was detected as a newly expressed protein upon Pb exposure (Figure 1c), thus providing another evidence for its role in heavy metal stress response.

RNA-binding proteins (RBPs) shuttle between cellular compartments either constitutively or in response to stress and regulate localization, translation, or turnover of mRNAs [29]. Post-transcriptional regulation can also occur through stabilization of mRNAs by specific RBPs in response to certain stimuli. The process of bulk export of mRNAs from nucleus to cytoplasm is highly conserved across eukaryotes. The export-competent mRNP consists of mRNAs and a dozen nucleocytoplasmic shuttling nuclear proteins, including RNA export factors, poly(A)-binding proteins, DEAD-box protein 5 and nucleoporins in yeast [30]. The RNA recognition motif (RRM) domain is by far the most abundant type of eukaryotic RNA-binding motif and besides mRNA binding, RRM domains involve in diverse protein-protein interactions. RBPs have been shown to translocate to the cytoplasm in response to stress. For example, A18 hnRNP, as induced by UV radiation, targets stress-activated transcripts and stimulates translation, thereby increasing survival after genotoxic stress [31]. In the present study, the identified RBPs were a newly-induced polyadenylate-binding protein (RRM superfamily) (Figure 1d) as well as two up-regulated proteins, namely splicing factor RNPS1 (Figure 1e) and ATP-dependent RNA helicase (Figure 1f). Splicing factor RNPS1 is a versatile splicing regulator for a wide variety of alternatively spliced genes and regulates alternative splicing both negatively and positively through interaction with associated factors in vivo [32]. Although their exact mechanism of function remains unclear, several ATP-dependent RNA helicases of the DEAD-box family have been described to be involved in transcription, pre-mRNA splicing, ribosome biogenesis, nuclear export, resolution of inhibitory mRNA secondary structures and translation initiation, RNA degradation and even organelle gene expression [33]. This enzyme was shown to be involved in adaptive response to oxidative stress in *Clostridium perfringens *[34], heat shock response in *Aspergillus fumigatus *[35] and various kinds of stress in many plants, including salt response in barley [36], pathogen infection and oxidative stress in transgenic *Arabidopsis *[37] and salt stress in the halophyte *Apocynum venetum *[38]. Taken together, up-regulation of above-mentioned proteins in *P. chrysosporium *in response to Pb stress strongly suggests that these proteins might act in concert to mediate transcriptional and post-tanscriptional regulations in a direction to overcome Pb toxicity.

An isoform of glyceraldehyde-3-phosphate dehydrogenase (GAPDH) (Figure 1g) was also among newly induced proteins in response to Pb. This extremely abundant glycolytic enzyme has multiple and unrelated functions. GAPDH expression was shown to increase in various organisms during apoptosis induced by a variety of stress factors. Potential role of its nuclear translocation in apoptosis and oxidative stress was proposed to be related with its activity as a DNA repair enzyme or as a nuclear carrier for pro-apoptotic molecules [39]. Different isoforms of GAPDH responding differently to H_2_O_2 _stress have been shown in *S*. *pombe *[40] and in budding yeast [41,42]. In *S*. *pombe*, peroxide stress signals are transmitted from the Mak2/3 sensor kinases to the Mpr1 histidine-containing phosphotransfer (HPt) protein and finally to the Mcs4 response regulator, which activates a MAP kinase cascade. Morigasaki *et al. *[43] recently showed that GAPDH plays an essential role in the phosphorelay signaling by physically associating with the Mcs4 response regulator and stress-responsive MAP kinase kinase kinases (MAPKKKs), where its redox-sensitive cysteine residue which is transiently oxidized in response to H_2_O_2 _stress may enhance its association. In *Arabidopsis thaliana*, the steady-state mRNA level of the cytosolic GAPDH increased when plants were transferred from normal growth condition to heat-shock, anaerobiosis, or increased sucrose supply [44] and the enzyme was recently shown to suppress heat shock-induced H_2_O_2 _production and cell death [45].

**Figure 2:**
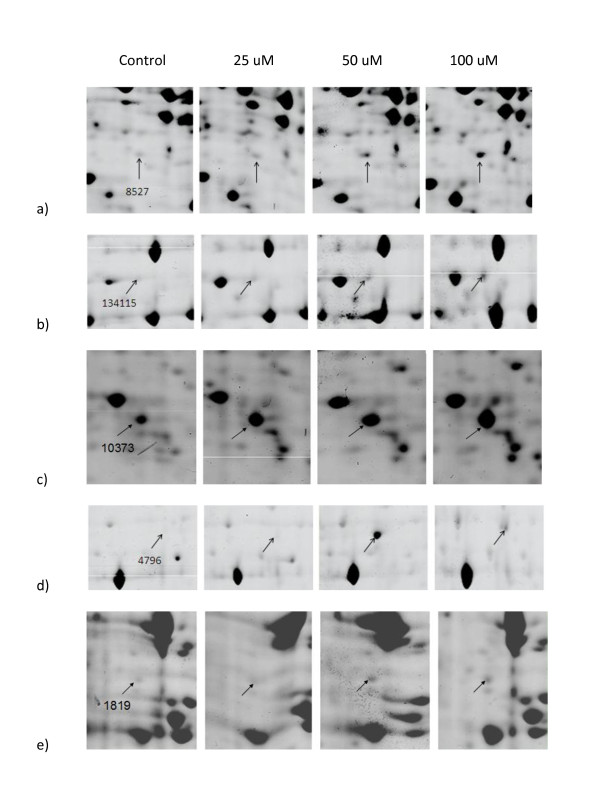
**Dose-dependent upregulated protein spots**.

The induction of redox enzyme thioredoxin reductase was quite expected given its role in the regeneration of reduced thioredoxin (Figure 2a). UDP-glucose is not only a necessary metabolite for cell wall biogenesis, but it is involved in the synthesis of the carbohydrate moiety of glycolipids and glycoproteins [46]. UDP-glucose pyrophosphorylase (Figure 2b) which is under the control of stationary phase transcription factor SigmaB in *B. subtilis *[47] was shown as a novel salt stress-responsive protein in rice [48]. G-protein-linked pathways evolved to allow responses to extracellular agonists in eukaryotic cells include those for nutrient sensing, pheromone response and mating, and pathogenesis in fungi [49], however except for the induction of Cd-induced G-protein β subunit in fission yeast [50], there is scarcity of literature reports on the roles of these pathways in stress response. On the other hand, evidence is accumulating for heterotrimeric G protein signaling in stress-associated physiological processes in plants. In mature leaves, G proteins transmit signals to molecules, including small GTPases, ion channels, and phospholipases which are the effectors in the responses to various stress conditions, including pathogens, ozone treatment and water deficit [51]. *Arabidopsis thaliana *with null mutation in the gene encoding β subunit of heterotrimeric G protein was more sensitive to O_3 _damage than wild-type plants [52]. The newly induced G protein beta subunit-like protein (Figure 2c) demonstrated in our work provided evidence for heterotrimeric G protein signaling under Pb(II) stress in *P. chrysosporium*.

Zn-containing alcohol dehydrogenase (Class V), with accession number 4796, gives the highest homology to *Cryptococcus neoformans *mannitol-1-P-dehydrogenase, a zinc-containing long chain alcohol/polyol dehydrogenase accumulating mannitol as an intracellular osmolyte and stress protectant [53]. Members of this family metabolize a wide variety of substrates, including ethanol, retinol, other aliphatic alcohols, hydroxysteroids, and lipid peroxidation products. The enzyme seemed to undertake one of the major functions to counteract with Cu toxicity [25] and Pb toxicity, as revealed by the present study (Figure 2d).

As to the down-regulated proteins, the most drastic effect was the decrease in abundance of certain isoforms of cobalamin-independent methionine synthase. Protein glutathionylation has been increasingly recognized as an important mode of regulation in eukaryotes and glutathionylation of key proteins involved in protein synthesis leads to inhibition of translation. MetE is so far one of the few proteins in bacteria known to be the most sensitive to oxidative damage. When stressed by an oxidant, glutathionylation of the active site of MetE protects the enzyme from permanent oxidative damage as shown in *E. coli *[54]. Thus, by turning off MetE in the face of oxidative stress, protein synthesis can be slowed or stopped, freeing cellular resources to be used elsewhere. Also, diminished expression of genes encoding enzymes in methionine and cysteine biosynthesis can be interpreted as a mechanism of securing sulfur for production of proteins involved in reductive detoxification, such as thioredoxin and glutathione [55].

Analysis of the proteome dynamics of mid-exponential phase cells of *P. chrysosporium *subjected acute lead exposure identified a total of 88 differentially expressed protein spots, 23 up-regulated and 67 down-regulated (Table 3 and 4). Regarding the up-regulated ones, seven proteins, namely aldehyde dehydrogenase, alcohol dehydrogenase class V, 60S acidic ribosomal protein P0 and particular isoforms of a putative protein, glyoxylate as well as certain isoforms of NAD-dependent malate dehydrogenase and glyceraldehyde-3-phosphate dehydrogenase were newly-induced upon lead exposure. For 17 out of a total of 23 up-regulated ones, the most significant increase was detected after 1 h exposure. Among the up-regulated spots identified, "energy production and conversion" proteins constituted the major KOG class as was found by Garcia-Leiro [56] in an analysis of oxidative stress response of *Kluyveromyces lactis*.

**Table 3:** Time-dependent upregulated protein spots in response to Pb

			Treatment/Control ratio					
KOG class	Protein ID	Putative Function	1h	2h	4h	8h	Subcellular location^*^	Multiple spots
**Amino acid transport and metabolism**	130118a	Glutamine synthetase	1,50	3,61	3,88	1,08	C	4
	139663	Methionine synthase II (cobalamin-independent)	3,51	0,58	2,29	1,58	C	3

**Carbohydrate transport and metabolism**	137211	Dihydroxyacetone kinase/glycerone kinase	9,99	3,50	3,37	3,93	C	-
	132198c	Glyceraldehyde 3-phosphate dehydrogenase	7,40	7,00	5,20	4,80	C	5
	132198d	Glyceraldehyde-3-phosphate dehydrogenase	new	new	new	new	C	5
	132198e	Glyceraldehyde 3-phosphate dehydrogenase	1,29	4,90	4,71	6,09	C	5

**Energy production and conversion**	133289	Aldehyde dehydrogenase	4,36	3,09	2,30	3,34	C	-
	134389	NADH-dependent flavin oxidoreductase/12-oxophytodienoate reductase	4,74	0,82	1,29	1,33	C	-
	138693	Aldehyde dehydrogenase	new	new	new	new	C	-
	123932a	Fumarate reductase, flavoprotein subunit	3,54	2,39	1,37	1,87	C	2
	129245a	NAD-dependent malate dehydrogenase	new	new	new	-	Mit	5
	133757c	Glyoxylate/hydroxypyruvate reductase (D-isomer-specific 2-hydroxy acid dehydrogenase superfamily)	new	new	new	new	C	3
	135576b	Inorganic pyrophosphatase/Nucleosome remodeling factor, subunit NURF38	1,24	1,34	8,02	0,84	Nuc	4

**General function prediction only**	10307	1,4-benzoquinone reductase-like; Trp repressor binding protein-like/protoplast-secreted protein	0,44	0,87	13,14	4,72	C	-
	132851	Glyoxylase	6,16	1,99	4,02	2,30	C	-

**NA**	140431a	Putative Protein	-	new	new	new	C	4
	140431b	Putative Protein	19,30	9,80	19,40	22,90	C	4
	140431c	Putative Protein	4,29	2,2	0,75	2,3	C	4

**Posttranslational modification, protein turnover, chaperones**	1827	20S proteasome, regulatory subunit alpha type PSMA6/SCL1	4,60	2,20	2,10	2,70	Mit	2

**RNA processing and modification**	8607	Splicing factor RNPS1, SR protein superfamily	3,32	3,12	0,95	3,5	Cyt_Nuc	-

**Secondary metabolites biosynthesis, transport and catabolism**	4796d	Alcohol dehydrogenase, class V	new	new	new	new	C	4
	127894	Zinc-containing alcohol dehydrogenase superfamily	5,21	3,22	5,32	6,55	C	-

**Translation, ribosomal structure and biogenesis**	130203	60S acidic ribosomal protein P0	new	new	new	new	C	-

*C; cytoplasmic, Ext; extracellular, Mit; mitochondrial, Nuc; Nuclear, Cysk; cytoskeleton, Cyto_nuc; cytoplasmic_nuclear

**Table 4:** Time-dependent downregulated protein spots in response to Pb

			Treatment/control ratio					
					
KOG Class	Protein ID	Putative Function	1h	2h	4h	8h	Subcellular Location*	Multiple spots
**Amino acid transport and metabolism**	137931	Glycine/serine hydroxymethyltransferase	0,56	0,27	0,19	0,42	C	3
	1542	3-isopropylmalate dehydrogenase	0,39	0,42	0,26	0,39	C	-
	10011	3-isopropylmalate dehydratase (aconitase superfamily)	0,15	0,55	0,14	0,18	Cysk	-
	125842	Lysine-ketoglutarate reductase/saccharopine dehydrogenase	0,43	0,17	0,08	0,33	C	-
	131837	Oxoprolinase	0,56	0,32	0,31	0,24	C	-
	134775	Glutamine amidotransferase/cyclase	0,53	0,67	0,59	0,26	C	-
	138721	Glutamate/leucine/phenylalanine/valine dehydrogenases	0,84	0,35	0,23	0,34	C	2
	139663	Methionine synthase II (cobalamin-independent)	0,25	0,36	0,05	0,17	C	5

**Carbohydrate transport and metabolism**	8743	Inositol monophosphatase	0,12	0,04	0,06	0,39	C	-
	10433	Transketolase	0,92	0,89	0,89	0,18	C	-
	133884	Glycolipid transfer protein	0,04	1,12	0,39	0,27	C	-
	137623	Phosphoglucomutase	1,54	0,35	0,17	1,07	C	-
	122435c	3-phosphoglycerate kinase	0,95	0,31	0,24	1,59	C	3
	3052a	Mannose-6-phosphate isomerase, type II	0,44	0,54	0,60	0,29	Cysk	2
	3052b	Mannose-6-phosphate isomerase, type II	0,91	0,44	0,18	0,32	Cysk	2

**Coenzyme transport and metabolism**	10308	S-adenosylhomocysteine hydrolase	0,67	0,64	0,30	0,56	C	-

**Cytoskeleton**	139298	Actin and related proteins	0,30	0,37	0,07	0,11	Cysk	4

**Defense mechanism**	10742	N-6 Adenine-specific DNA methylase	1,05	0,6	0,32	0,19	Mit	-

**Energy production and conversion**	912	Kynurenine 3-monooxygenase and related flavoprotein monooxygenases	0,78	0,41	0,19	0,20	Ext	-
	1350	Aldehyde dehydrogenase	0,45	0,34	0,36	0,29	C	-
	132162	NAD-dependent malate dehydrogenase	0,00	0,37	0,55	0,51	C	-
	132918	Sulfide:quinone oxidoreductase/flavo-binding protein	0,85	0,49	0,22	0,55	Mit	-
	134368	Vacuolar H+-ATPase V1 sector, subunit E	0,29	0,67	0,32	1,98	Nuc	-
	135659	NADP+-dependent malic enzyme	1,13	0,72	0,18	1,18	Mit	-
	140211	Glyoxylate/hydroxypyruvate reductase (D-isomer-specific 2-hydroxy acid dehydrogenase superfamily)	1,51	0,51	0,29	0,33	C	-
	123932b	Fumarate reductase, flavoprotein subunit	1,21	0,85	0,60	0,10	C	2
	131257a	NADH-ubiquinone oxidoreductase, NDUFS1/75 kDa subunit	0,91	0,88	0,42	0,21	C	3
	131879a	Dihydrolipoamide dehydrogenase	0,62	0,52	0,26	0,80	C	2
	133924a	Aldehyde dehydrogenase	0,65	0,49	0,26	0,61	C	2
	133924b	Aldehyde dehydrogenase	0,31	0,48	0,28	0,55	C	2
	563a	Pyruvate dehydrogenase E1, alpha subunit	1,33	0,28	0,27	0,28	Mit	2

**General function prediction only**	3442	Predicted NAD-dependent oxidoreductase	0,30	0,22	0,54	0,50	C	-
	132767b	Serine/threonine protein kinase, active site	0,55	0,85	0,55	0,27	Mit	2

**Lipid transport and metabolism**	511	Enoyl-CoA hydratase	1,04	0,74	0,14	0,96	C	-
	10355b	Mevalonate pyrophosphate decarboxylase	0,73	0,36	0,24	0,48	Mit	2

**Posttranslational modification, protein turnover, chaperones**	361	26S proteasome regulatory complex, ATPase RPT3	0,25	0,17	0,06	0,05	Nuc	-
	1324	Molecular co-chaperone STI1	1,02	0,68	0,01	1,11	C	-
	1846	Chaperonin complex component, TCP-1 beta subunit (CCT2)	0,79	0,85	0,46	0,28	C	-
	5061	Dipeptidyl aminopeptidase	1,06	1,07	0,99	0,29	Ext	2
	8527	Thioredoxin reductase	0,27	0,45	0,22	0,31	Mit	-
	130274	Glutathione peroxidase	0,33	0,77	1,10	0,19	C	-
	131571	Protein disulfide isomerase (prolyl 4-hydroxylase beta subunit)	0,91	0,08	0,74	0,06	Ext	-
	133185	26S proteasome regulatory complex, ATPase RPT1	0,31	0,34	0,07	0,04	Nuc	-
	133717	20S proteasome, regulatory subunit alpha type PSMA4/PRE9	0,30	0,62	0,82	0,73	Mit	-
	134073	Chaperonin complex component, TCP-1 zeta subunit (CCT6	0,70	0,42	0,14	0,10	C	-
	139500	Multifunctional chaperone (14-3-3 family)	0,27	0,38	0,20	0,47	Nuc	-
	10340a	HSP70(putative ortholog to S. cerevisiae Heat shock protein homolog SSE1 (Chaperone protein MSI3)	0,66	0,54	0,06	0,64	C	2
	10340b	HSP70(putative ortholog to S. cerevisiae Heat shock protein homolog SSE1 (Chaperone protein MSI3)	0,34	0,15	0,20	0,26	C	2

**Secondary metabolites biosynthesis, transport and catabolism**	133231	Predicted dehydrogenase	0,26	0,53	0,16	0,10	C	-
	4796b	Alcohol dehydrogenase, class V	0,61	0,31	0,17	0,07	C	4

**Translation, ribosomal structure and biogenesis**	3216	Prolyl-tRNA synthetase	0,80	0,60	0,51	0,12	C	-
	6570	Mitochondrial translation elongation factor Tu	0,61	0,59	0,16	0,76	Mit	-
	10819	Elongation factor 2	1,27	0,65	0,15	0,14	C	-

*C; cytoplasmic, Ext; extracellular, Mit; mitochondrial, Nuc; Nuclear, Cysk; cytoskeleton, Cyto_nucl; cytoplasmic_nuclear

**Figure 3:**
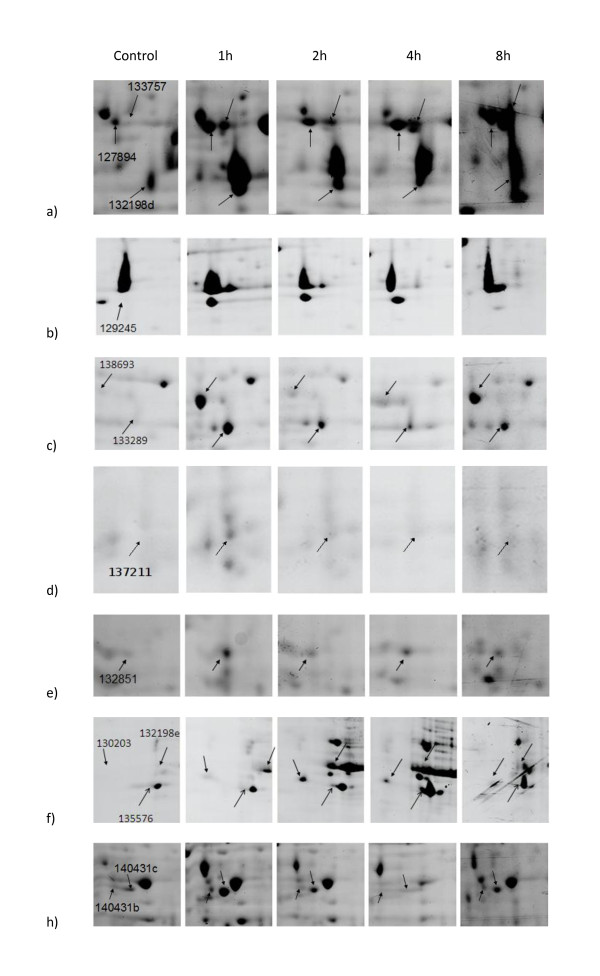
**Time-dependent upregulated protein spots**.

Although Pb (II) is not a redox active metal ion, it is known to cause oxidative stress resulting in increased production of reactive oxygen species (ROS) inducing lipid peroxidation [57,58]. There were several newly induced/strongly up-regulated proteins involved in amelioration of lipid peroxidation products, namely zinc-containing alcohol dehydrogenase, glyceraldehyde-3-phosphate dehydrogenase, glyoxylate/hydroxypyruvate reductase (Figure 3a) and two different aldehyde dehydrogenases (ALDH; Figure 3c) detected after 1 h exposure. NAD-dependent malate dehydrogenase (MDH; Figure 3b), on the other hand, is a very well known up-regulated component of different stress conditions in various organisms. It provides protection against oxidative damage caused by Zn in *E*. *coli *through the action of oxaloacetate [59]. In the fission yeast, MDH was one of the component core environmental stress response (CESR) as determined by transcriptional profiling, proteomic and metabolomic analysis [40,50]. The other components of CESR included zinc-binding dehydrogenases, serine/threonine protein kinase, G-protein beta subunit, quinone oxidoreductase, flavin oxidoreductase, protein with RNA recognition motif and short chain dehydrogenase, all were found to be up-regulated in *P. chrysosporium *cells in our former [25] and present study. Hot pepper transcriptome profiling under cold stress [60] proteomic analysis of Cd response in marine alga *Nannochloropsis oculata *[61] DNA microarray and quantitative RT-PCR analyses in *Corynebacterium glutamicum *under oxygen deprivation [62], analysis of a metabolic network in *Pseudomonas fluorescens *exposed to oxidative stress [63] and transcriptomic analysis of Al stress in roots of *Arabidopsis thaliana *[64] were among other studies consistently detecting MDH involvement. As discussed by the latter authors, the burst of ROS generated by Al had to enhance the generation of NADPH to maintain a high ratio of reduced antioxidants.

In the present study, two different ALDHs with the protein IDs of 133289 and 138693 were found to display a biphasic up-regulation (at 1 h and 8 h) upon Pb treatment (Figure 3c). ALDH superfamily enzymes and their pathophysiological significance was recently reviewed by Marchitti *et al. *[65]. Induction of ALDH in response to oxidative stress was demonstrated also in bacteria, e.g. *Pseudomonas aeruginosa *[66] and yeast [67]. As to the plants, overexpression of ALDH3 genes in *A*. *thaliana *confers tolerance to various abiotic stress conditions and protects plants against lipid peroxidation [68,69].

The expression of remarkably many genes encoding proteins and enzymes involved in defense from oxidative damage and redox metabolism is stimulated under a range of stress conditions. Examples of such genes include catalase, thioredoxin, glutaredoxin, genes encoding enzymes possibly involved in detoxification such as glyoxylase and dihydroxyacetone kinase, and numerous oxidoreductases that may be involved in metabolism of oxidized biomolecules or in adjusting redox metabolism to provide sufficient NADPH for detoxification. Dihydroxyacetone kinase/glycerone kinase (DAK) was induced in *P. chrysosporium *1 h after Pb administration (Figure 3d). Its relation with salt stress [70], heat stress [71], osmotic stress [72], oxidative stress [73], starvation [74] and cadmium stress [21] was well documented. Glyoxylase system detoxifying glyoxal, methylglyoxal and other physiological alpha-oxoaldehydes formed by lipid peroxidation is a component of stress response, as shown for yeasts [75,76], various plants [77-79], a parasitic nematode [80] and in a basidiomycete, as shown by our study (Figure 3e).

Inorganic phosphatase constituties NURF-38 subunit of the ATP-dependent Nucleosome Remodeling Factor complex (NURF) which was initially identified in *Drosophila *and then in yeast and vertebrates [81,82]. The complex, when targeted onto chromatin, affects major DNA-dependent processes including transcription, DNA repair and recombination. Thus, our finding that inorganic pyrophosphatase/nucleosome remodeling factor NURF38 is induced by a factor of 8 upon 4 h lead exposure pointed to its role in responding to metal toxicity in *P. chrysosporium *(Figure 3f). The 60S acidic ribosomal protein P0 was one of the newly-induced component of Pb (II)-stressed proteome. This protein plays an essential role by docking and forming the tip of the whole eukaryotic ribosomal "stalk" complex [83]. Unlike the typical ribosomal proteins, P0 appears to have multiple functions in the cell. By overexpressing *Drosophila *P0 in *E*. *coli*, Yacoub *et al. *[84] demonstrated that P0 contained 5' APE activity distinct from the 3' AP lyase activity associated with *Drosophila *rpS3 and also exhibited nuclease activity against both double and single-stranded DNA. The authors also reported that the protein is located in both nucleus and ribosomes. Recently, this protein was shown to be upregulated in Ras-transformed NIH3T3 cells [85] and Jurkat cells during heat stress-induced apoptosis [86]. Additionally, when the *S. pombe *cells challenged with oxidative stress, 60S ribosomal protein P0 was among differentially-expressed ones though its induction ratio was only 1.6 [40] (Figure 3f).

Being consistent with our former report on Cu and Cd response of the organism [25], the downregulated proteins included some redox enzymes and certain molecular chaperons like HSP70, protein disulfide isomerase and chaperonin complex components.

Bacteria and fungi are among the first components of the biota in ecosystems affected by toxic pollutants including heavy metals. Their relatively small size and simplicity make them particularly attractive models for environmental proteomics [18]. The data obtained from the analysis of proteomes of such organisms help to gain insight into underlying mechanisms of toxicity which is of great value from the basic sciences pointview. Besides, the subtle changes detected in the level of individual proteins in response to environmental stressors lead to the discovery of biomarkers of exposure and also provide an opportunity to genetically engineer such microbes to express higher levels of specific proteins (e.g. DNA repair proteins), thereby conferring higher tolerance to increasing concentrations of heavy metals and potentiate bioremediation [87,88]. Our studies are under way to obtain complete coverage of the lead-induced stress proteome of *P. chrysosporium*, hence identifying all possible ecotoxicological biomarkers as well as targets for improved lead bioaccumulation.

The apoptotic machinery in fungi was recently reviewed by Sharon *et al. *[89]. Among fungi, apoptosis has only been studied in detail in *S. cerevesiae*. Filamentous fungal species are poorly analyzed for functions of the apoptosis-related genes and although homologs of some apoptotic genes could be identified in fungal genomes analyzed, to date only a few genes have been functionally analyzed. A better understanding of fungal apoptotic networks for identification of paralogs and putative homologs of apoptosis as compared to those of high eukaryotes is required which is possible through findings from more fungal species. As reported by Lorin *et al. *[90] for the filamentous fungus *Podospora anserina*, compensatory induction of an alternative oxidase (AOX) provides a decreased production of ROS and a striking increase in lifespan. In the present research, the elements like actin known to induce hyperactivation of the Ras signaling pathway, septin family protein (P-loop GTPase) functioning in many processes including apoptosis, Ras GTPase of acceleration of programmed cell death are identified for the first time in a multicellular fungus and expected to contribute to the knowledge on stress-induced apoptosis in fungi. When exposed to lead stress, the elements of stress tolerance and initiation of apoptotic cell death counteracted while the cells of *P. chrysosporium *kept on growing. On the other hand, our findings on induction/upregulation of certain catabolic enzymes and mitochondrial F0F1-type ATP synthase might provide support for an intrinsic and mitochondrial nature of the apostatic response in fungi and fit to the generally accepted view that apoptosis requires energy as it is a highly regulated process involving a number of ATP-dependent steps [91].

## Conclusion

The present study draws attention particularly to the up-regulated elements of apoptosis, DNA repair, post-tanscriptional regulation and heterotrimeric G protein signaling as shown for the first time for a metal-stressed basidiomycete. DNA-binding response regulator(s) mediating stress response in *P. chrysosporium*, like Yap1p TF and Yap2p TF of *S. cerevisiae *[92], Sty1p-activated Atf1p and Pap1p TF of the fission yeast [50,93] and Cap1p TF of *Candida albicans *[76] remains to be identified through further analysis.

## Materials and methods

### Culture conditions

*P. chrysosporium *(ATTC 24725) spores were separated from Sabaroud Dextrose agar slant surfaces by scrapping, homogenized and suspended in sterile distilled water. A spore suspension was prepared to contain 2.5 × 10^6 ^spores.mL^-1 ^at an absorbance of 0.5 at 650 nm using a Shimadzu UV-1208 spectrophotometer. The suspension was then transferred into a 250 mL Erlenmayer flasks each containing 150 ml of the growth medium described by Prouty [94] which was composed, in g.L^-1^, of glucose, 10; KH_2_PO_4_, 2; MgSO_4_, 0.5; CaCl, 0.1; NH_4_Cl, 0.12 and thiamine, 0.001 and adjusted to a pH of 4.5. The cultures were incubated for 40 h at 200 rpm in a rotary shaker at 35°C. When the growth was terminated at 40^th ^h, the cultures were still in exponential growth.

For proteomic characterization of the lead response, the cells of *P. chrysosporium *were grown in minimal media containing different levels of lead (25, 50 and 100 μM Pb(NO_3_)_2_, respectively) for 40 h till they reached mid-exponential phase and they were harvested by filtration, washed twice with distilled water and stored at -20°C for the extraction of proteins. As a parallel setup, *P. chrysosporium *cells were grown on minimal media for 40 h till they reached mid-exponential phase and then subjected to 50 μM lead for 1, 2, 4 and 8 h to investigate the effect of duration of temporal Pb (II) exposure on *P. chrysosporium *proteome. For both cases, a control sample, without lead exposure, was harvested at 40^th ^h of the growth.

### Protein Extraction

After crushing the cells with liquid nitrogen, TCA-acetone extraction was performed as in Damerval *et al. *[95]. After breaking a 500 mg of harvested mycelium in liquid nitrogen, 5 mL of 10% trichloroacetic acid in acetone containing 0.07% β-mercaptoethanol was added and vortexed, then incubated at -20°C for 45 min and centrifuged at 15 000 *g *for 15 min. The supernatant was decanted and the pellet was resuspended in 5 mL of acetone containing 0.07% β-mercaptoethanol which was then incubated at -20 °C for 1 h (mixed every 15 min intervals by vortexing) and recentrifuged. The supernatant was discarded, the remaining pellet was vacuum-dried and stored as a powder at -20°C. The modified Bradford assay [96] was used to determine protein concentrations.

### 2DE

2D gels of the harvested cells were run in duplicates for control and each treatment. Isoelectric focusing was performed in 18 cm linear IPG-strips (pH range 3-10, Biorad, Hercules, CA, USA). IPG strips were passively rehydrated by applying 300 μl of rehydration buffer containing 8 M urea, 2 M thiourea, 1% w/v CHAPS, 20 mm DTT and 0,5% v/v ampholyte 3-10 with 300 μg protein sample for 16 h. Isoelectric focusing was performed with the Protean IEF Cell unit (Biorad, Hercules, CA, USA) employing a total of 80 000 Vh. After consecutive equilibration of the gels in solutions containing DTT and iodoacetamide as suggested by Görg *et al. *[97], the separation in the second dimension was done in polyacrylamide gels of 12.5% T and 2.6% C on the Biorad Protean Xii electrophoresis system (Biorad, Hercules, CA, USA) by applying 2 W per gel. Gels were stained with colloidal Coomassie blue [98].

### Image analysis

Coomassie stained gels were digitized by using an HP scanner. Spot pattern analyses were accomplished by using the 2D image analysis software Delta2D version 3.3 (Decodon, Germany). Of the proteins found differentially expressed in Pb (II)-exposed cells, only those showing at least 3 fold difference in abundance were selected and subjected to MALDI-TOF analysis. To correct the quantitative variability, the spot volumes were normalized as a percentage of the total volume in all of the spots in the gel. SDs of the spot intensities from the two replicates were in the range of 20%.

### Protein identification

The identifications were accomplished by mass spectrometry according to established protocols. Briefly, protein spots were excised from stained 2D gels, destained and digested with trypsin (Promega, Madison, WI, USA) and for extraction of peptides, the gel pieces were covered with 60 μl 0.1% trifluoroacetic acid in 50% CH_3_CN and incubated for 30min at 40°C. Peptide solutions were mixed with an equal volume of saturated α-cyano-3- hydroxycinnamic acid solution in 50% acetonitrile-0.1% trifluoroacetic acid (v/v) and applied to a sample plate for MALDI-TOF-MS. Mass analyses were carried out on the Proteome-Analyzer 4700 (Applied Biosystems, Foster City, CA, USA). The three most abundant peptides in each MS spectrum were chosen for MS/MS experiment. The resulting sequence data were included for the database search to increase the reliability of protein identification. Mass accuracy was usually in the range between 10 and 30 ppm.

### Database searches

Amino acid sequences for *P. chrysosporium *proteins were obtained from organism's genome project [Joint Genome Institutes (JGI)] web site [99]. PMF and MS/MS data was searched in the *P. chrysosporium *data with the aid of MASCOT software [100]. The searches considered oxidation of methionine and modification of cysteine by carbamidomethylation as well as partial cleavage leaving one internal cleavage site. Of the results given by the MASCOT software, those having a probability score value higher than 53 were considered for successful protein identification. To find out putative functions, protein accession numbers of the identified spots were searched in the JGI website for P. chrysosporium. For the identified proteins, the functional classification was made by consulting to the functional categories list contained in the same website.

Protein subcellular localization predictions were obtained from WoLF PSORT web server [101].

## Competing interests

The authors declare that they have no competing interests.

## Authors' contributions

VY carried out the 2DE experiments for time dependent Pb exposure studies. SÖ carried out the 2DE experiments for dose dependent Pb exposure studies. DB carried out the mass spectrometry analyses and protein identifications. KB participated in the optimization of 2DE protocol for P. chrysosporium. MH participated in the design of the study. GÖ conceived of the study, and participated in its design and coordination. All authors read and approved the final manuscript.
